# First report on isolation of *Mycobacterium monacense* from sputum specimen in India

**DOI:** 10.4103/0970-2113.80326

**Published:** 2011

**Authors:** K. Lily Therese, R. Gayathri, K. Thiruppathi, H. N. Madhavan

**Affiliations:** *L & T Microbiology Research Centre, Vision Research Foundation, Chennai, India*; 1 *Narayana Hospitals, Chennai, India*

**Keywords:** *Mycobacterium monacense*, rapid growers of mycobacteria, sputum

## Abstract

We report a case of *Mycobacterium monacense* (*M. monacense*) isolated from sputum of a female patient for the first time in India. The chest radiograph and symptoms were suggestive of pulmonary tuberculosis. *M. monacense* was isolated from the sputum specimen at the end of 11 days of incubation. The identification was confirmed by conventional biochemical tests and polymerase chain reaction (PCR) -based Restriction Fragment Length Polymorphism (RFLP) and DNA sequencing targeting Internal Transcribed Spacer (ITS) region and *hsp65* gene. The patient was treated with conventional anti-tuberculous drugs.

## INTRODUCTION

Rapid growers of mycobacteria (RGM) are ubiquitous in nature, and currently there are more than 153 species of mycobacteria known to be pathogenic to human beings. The pathogenic role of RGM has been underestimated for several years. Only in recent years, there have been many reports on the association of RGM with a variety of clinical conditions. This is the first report on isolation of *Mycobacterium monacense* associated with pulmonary tuberculosis-like disease in India.

## CASE REPORT

**Figure 1:**
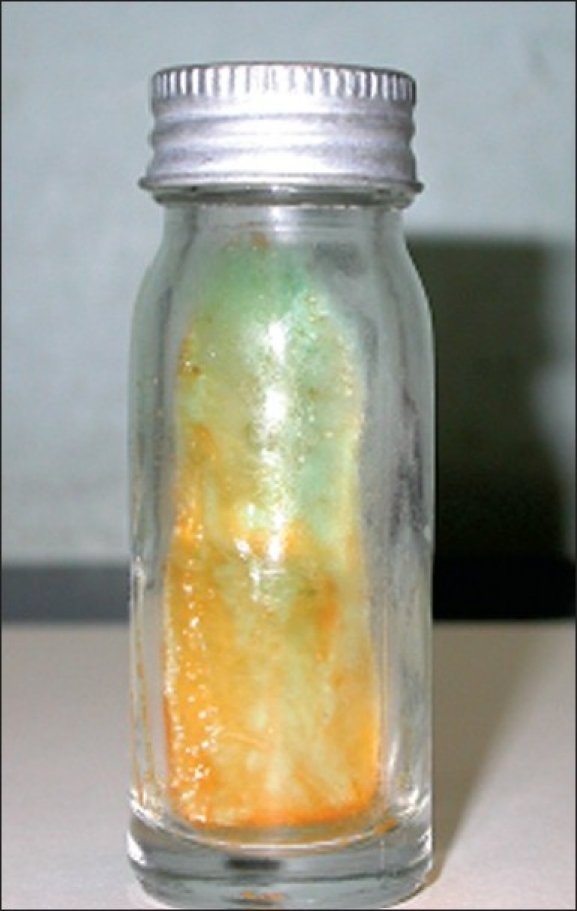
Dark yellow pigmented colonies of *M. monacense* in Lowenstein-Jensen (LJ) medium

**Figure 2:**
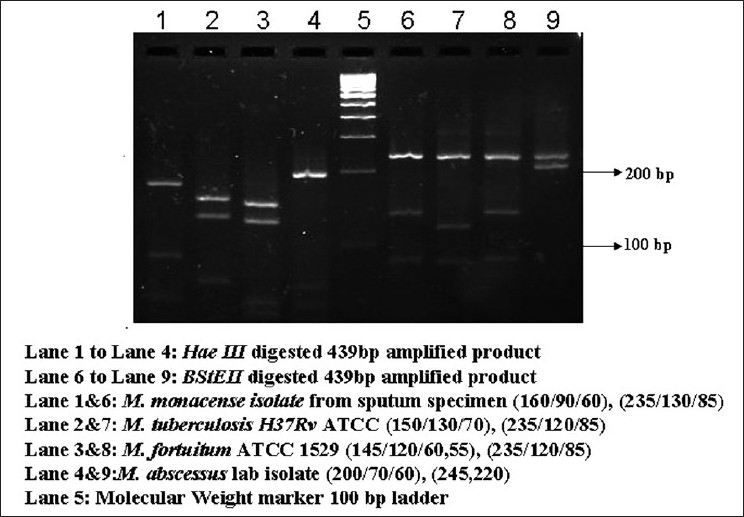
PCR RFLP of *M. monacense* with HaeIII and BstEII

**Figure 3:**
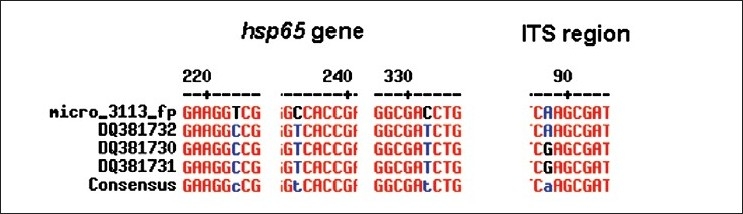
Alignment of nucleotide serovars found in the *hsp65* gene and Internal Transcribed Spacer (ITS) region of published sequences and the new isolate

The patient was a 43-year-old HIV-negative female, a known diabetic. The patient visited a private clinic in Chennai, India, with complaints of intermittent coughing and respiratory problems, along with loss of appetite. The laboratory investigations showed increased ESR, elevated alkaline phosphatase level, calcification in liver; and chest imaging showed multiple air spaces with nodules. The sputum specimen of this patient was sent to our laboratory for mycobacteriological tests, the direct smear for acid-fast bacilli and culture by BACTEC method. The Ziehl-Neelsen staining performed on the direct and concentrated smear of the sputum was negative for Acid-Fast Bacilli. Nested polymerase chain reactions (nPCRs) targeting *MPB64* gene[1] and IS6110[2] region for detection of *M. tuberculosis* genome were also negative. The sputum specimen was inoculated in BACTEC 12B medium after performing the standard decontamination procedure with NALC (N-acetyl L-Cysteine)-NaOH (Becton Dickinson) as per the manufacturer’s instructions. At the end of 11 days, BACTEC TB 460 reader showed a positive growth index. nPCRs targeting *MPB64* gene and IS6110 region of *M. tuberculosis* on the isolate were also negative, indicating that the isolate was nontuberculous mycobacterium. The isolate was acid fast and scotochromogenic [Figure 1]. It took 5 days to grow on Lowenstein-Jensen medium. It grew on blood agar but not on MacConkey agar. The conventional biochemical and other tests performed for identification showed positive results for growth in 4.5% NaCl, Iron uptake test, Nitrate reductase test, Thermostable catalase test and Tween-80 hydrolysis test; and negative results for Niacin accumulation test and Aryl sulfatase production test. The isolate was identified as *Mycobacterium monacense*. In order to confirm the identification, PCR-based RFLP and DNA sequencing targeting *hsp65*[3] gene and PCR-based DNA sequencing targeting Internal Transcribed Spacer (ITS)[4] region were performed for the isolate. PCR-based RFLP[3] using *BstEII* and *HaeIII* was performed, which yielded fragments 235, 130, 85; and 160, 90, 60; respectively [Figure 2]. The RFLP products matched with the RFLP pattern of *M. monacense* reported earlier,[5] confirming the phenotypic identification. The results of PCR-based DNA sequencing of both the targets also revealed 98% homology with *M. monacense* reference strains deposited in the Genbank[6] (DQ381730, DQ381731, DQ381732 for hsp65; and DQ466897, DQ466902, DQ466898 for ITS). The nucleotide sequence of the *M. monacense* isolate was deposited in Genbank and given the accession nos. FJ59604, FJ59605, FJ59606, FJ59607. Multalin analysis revealed that the isolate was in consensus with the already reported sequences. [Figure 3].

Antibiotic susceptibility testing was performed by BACTEC TB 460 reader for the first-line drugs (streptomycin, isoniazid, rifampicin, ethambutol and pyrazinamide); and by Kirby Bauer method for the following antibiotics[7]: cefoxitin, doxycycline, amikacin, azithromycin, tobramycin, clarithromycin, ciprofloxacin, ofloxacin, norfloxacin, gatifloxacin and moxifloxacin.

## DISCUSSION

There are 5 case reports till date in literature on the isolation of *M. monacense* associated with human infections mainly isolated from respiratory specimens[6,8]. The first strain was isolated in 1998 in Germany from a bronchial lavage of an 80-year-old patient hospitalized for treatment of multifocal lung carcinoma and insulin-dependent diabetes mellitus.[6] Three other strains were isolated between 2000 and 2005 from unrelated patients hospitalized in different cities in Italy. The second strain was isolated from the biopsy material of an 11-year-old boy presenting with a fistula on his right thigh. The third and fourth strains were grown from the sputum of a 31-year-old HIV-positive woman with bronchopneumonitis and from the sputum of an 82-year-old patient suspected to have lung cancer, respectively.[6] The fifth strain was isolated from broncho-alveolar lavage of a 36-year-old man with pulmonary tumor.[8]

*M. monacense* isolated in the study exactly matched with the textbook description and with the other case reports in literature[6,8]. The susceptibility of the isolate to the first-line anti-tuberculosis drugs was also determined by BACTEC TB 460 reader since the patient was treated empirically with first-line anti-tuberculosis drugs. The isolate was sensitive to streptomycin, isoniazid, rifampicin, ethambutol, pyrazinamide, cefoxitin, doxycycline, amikacin, azithromycin, tobramycin, clarithromycin, ciprofloxacin, ofloxacin, norfloxacin, gatifloxacin and moxifloxacin.

The patient was lost for follow-up. However, the underlying cause of the disease was not completely understood. The limitation of the study was that repeat specimen could not be collected from the same patient to confirm the result.

## CONCLUSION

To the best of our knowledge, this is the first case report of isolation of *M. monacense* from sputum in India. This case suggests that *M. monacense* should be considered to be a potential human pathogen associated with pulmonary disease. Further studies are required to evaluate the pathogenic potential of *M. monacense* for humans.
